# Acute effects of coffee consumption on self-reported gastrointestinal symptoms, blood pressure and stress indices in healthy individuals

**DOI:** 10.1186/s12937-016-0146-0

**Published:** 2016-03-15

**Authors:** Emilia Papakonstantinou, Ioanna Kechribari, Κyriaki Sotirakoglou, Petros Tarantilis, Theodora Gourdomichali, George Michas, Vassiliki Kravvariti, Konstantinos Voumvourakis, Antonis Zampelas

**Affiliations:** 1 Department of Food Science and Human Nutrition, Agricultural University of Athens, IeraOdos 75, Athens, 11855 Greece; 2 Department of Crop Science, Agricultural University of Athens, Athens, Greece; 3 Second Department of Neurology, University of Athens Medical School, “Attikon” University Hospital, Athens, Greece; 4 Department of Nutrition and Health, United Arab Emirates University, Al Ain, United Arab Emirates

**Keywords:** Coffee, Salivary alpha-amylase, Salivaly cortisol, Stress, Blood pressure

## Abstract

**Background:**

It has been suggested that coffee may affect the gut-brain axis with conflicting outcomes. Moreover, there is insufficient evidence to determine whether the type or temperature of coffee consumed will have a different impact on the gut-brain axis. The purpose of this study was to investigate the effects of acute coffee consumption on the following: 1. self-reported GI symptoms and salivary gastrin, 2. stress indices [salivary cortisol and alpha-amylase (sAA)] and psychometric measures, and 3. blood pressure (BP), in healthy, daily coffee consuming individuals in non-stressful conditions.

**Methods:**

This was a randomized, double blind, crossover clinical trial, in which 40 healthy individuals (20 men, 20 women), 20–55 years of age, randomly consumed four 200 ml coffee beverages containing 160 mg caffeine (hot and cold instant coffee, cold espresso, hot filtered coffee), 1 week apart. Salivary samples and psychometric questionnaires were collected at baseline and post-coffee consumption at 15,30, and 60 min for salivary gastrin and sAA measurements and at 60,120, and 180 min for cortisol measurements. BP was measured at beginning and end of each intervention. *ClinicalTrials.gov ID: NCT02253628*

**Results:**

Coffee consumption significantly increased sAA activity (*P* = 0.041), with significant differences only between cold instant and filter coffee at 15 and 30 min post-consumption (*P* < 0.05). Coffee temporarily increased salivary gastrin, without differences between coffee types. Coffee did not affect salivary cortisol or self-reported anxiety levels. Coffee consumption significantly increased BP, within the healthy physiological levels, in a gender specific manner at the end of the experimental periods, without differences between coffee types.

**Conclusion:**

Acute coffee consumption in non-stressful conditions activated sAA and BP but not salivary cortisol, indicating activation of the sympathetic nervous system. Post-coffee sAA increase without a concomitant cortisol increase may also indicate that coffee may have some anti-stress properties.

## Background

Coffee is a widely consumed, pharmacologically active beverage, which has been suggested to affect the gut-brain axis with conflicting outcomes. The gut-brain axis affects a number of physiological processes that result in satiety and food intake as well as wellbeing and mood and may also relate to gastrointestinal (GI) function and/or GI disturbances [1, 2]. Visualization of food, anticipation of eating and perceived stress [3] initiate profound changes in digestive function that are characterized by the release of a variety of GI hormones, including gastrin, which lead to gastric acid secretion [1]. In some studies, the coffee-mediated increased gastric acid secretion [4] has been associated with GI disturbances, such as gastroesophageal reflux disease (GERD), epigastric pain, reflux, and heartburn [5]. However, results from clinical and epidemiological trials have shown that coffee consumption has protective effects on GI function with no associations with gastric ulcer, duodenal ulcer, reflux esophagitis, reddish streaks, and non-erosive reflux disease [6–8]. Furthermore, the stimulation and interrelation of the gut-brain axis may explain why some people with a healthy, functioning GI system experience symptoms such as increased anxiety, abdominal pain or discomfort, and GERD upon nutrient or coffee ingestion; these symptoms cannot be medically explained and are unrelated to coffee consumption and overall dietary pattern [1, 3, 9–11].

Gastrin, which is secreted in response to a meal [12] and can be measured in saliva [13], thus allowing it to be evaluated in non-stressful conditions, is one of the hormones regulating the rhythm of gastric acid secretion [14]. Some studies [15–19], but not all [18, 20, 21], have shown that coffee consumption (independently of caffeine content) increases gastrin secretion, regardless of coffee temperature (cold, warm, hot) [22].

Some studies [23–26], but not all [27–29], have suggested that caffeinated coffee may activate the hypothalamic-anterior pituitary-adrenocortical (HPA) axis, increasing the secretion of stress hormones, i.e., cortisol, in a gender-dependent manner [30], as well as temporarily increasing blood pressure (BP), even in small caffeine doses [31], and basal metabolic rate [32]. It has been reported that from all the GI hormones involved in gastric acid secretion, only gastrin concentrations follow the behavior of cortisol; because both hormones increase after the physiological stress experienced by athletes during different types of competitions without, however, reaching the levels reported for GI pathologies [33].

Moreover, a few studies [34], but not all [35], have suggested that caffeinated coffee may activate the sympathetic nervous system (SNS), via secretion of salivary alpha-amylase (sAA) [36–40], an enzyme involved in polysaccharide digestion [41]. sAA is a convenient marker and is easily collected and measured in saliva [36, 42–44]; it is recognized as both a soothing/relaxation index [45] and a stress response marker [36].

Therefore, due to the limited and contradictory findings of the literature, the purpose of this study was to examine the effects of acute consumption of different types and temperature of coffee (hot and cold instant coffee, cold espresso and hot filtered coffee), containing equal amounts of caffeine in non-stressful conditions on 1. self-reported GI and salivary gastrin; 2. stress indices (salivary cortisol, sAA and self-reported anxiety levels); and 3. BP in healthy men and women. The study is the first such examination, to our knowledge, and was performed in the context of the limited and contradictory findings concerning coffee consumption and the gut-brain interrelationship.

## Methods

### Subjects

Forty healthy volunteers (20 men and 20 women), 20–55 years old, with body mass index (BMI) ≥18.5 and <27 kg/m^2^, who consumed coffee daily and did not object to consuming cold coffee, participated in this randomized, double blind, crossover study. Volunteers had to have stable body weight for at least 1 month before entering the study. Exclusion criteria included consuming breakfast >3 days/week (so that it would not be uncommon practice for the volunteers to consume coffee without food and remain on an empty stomach for many hours), daily intake of ≥500 mg caffeine, a psychological state strongly influenced by outside factors (e.g. moving to new home, work change, separation from a loved one, etc.), attempting to lose weight at the time of the study, smoking, intense physical activity (>4 h per day), medical/drug therapy, depression, medical conditions (i.e., diabetes mellitus, hypertension, and similar factors).

All volunteers signed a consent form and the research protocol was approved by the Bioethics Committee of the Agricultural University of Athens. The study was completed at the Human Nutrition Unit of the Department of Food Science and Human Nutrition of Agricultural University of Athens.

### Experimental procedure

All studies started between 08.30 and 09.00 h. All volunteers completed a medical and demographic questionnaire on their initial visit. Each volunteer attended four separate experimental sessions, 1 week apart. For allocation of the participants, a computer-generated list of random numbers was used (http://www.random.org/sequences/). One person, not involved in data handling and analysis, was responsible for the test coffee allocation. The coffee was served in a paper cup covered with a lid, and neither the participants nor the investigators were aware of the sequence or the content of paper cups. The crossover design was selected to limit the inter-subject variability between the interventions [46].

At each experimental session, 200 ml of coffee beverage was consumed and contained 160 mg caffeine and 7.5 g white sugar: [1. hot instant coffee (Nescafé Classic, Nestlé); 2. cold instant coffee (Nescafé Classic, Nestlé); 3. cold espresso (Buondi prestige, Nestlé); and 4. hot filtered coffee (Nestlé Professional Plantage Filter Coffee, Nestlé]. Volunteers came to the Human Nutrition Unit at the same time and day of the week, ruling out the possibility of confounding effects of different days of the week (i.e. Friday may be a less stressful day than Monday for some people). The day preceding the test coffee, the volunteers were required to a) not consume anything containing caffeine, thus ruling out additive results if the previous day’s caffeine had been not fully metabolized, b) not consume alcohol, thus limiting the possibility of dehydration status effects [47], c) avoid exercise, thus reducing the possibility of dehydration status effects [48], d) get adequate sleep the previous night (~7 h), e) fast for 8 h, f) consume approximately the same foods the day before the test as consumed on the day preceding the 1^st^ test beverage, in the same quantities and times of day, thus preventing confounding effects from the previous day’s dietary intake. To accomplish this, a photocopy of the baseline 24 h recall obtained was given to each volunteer. Volunteers were not allowed to perform physical activity or consume food/liquids during the study and were asked to remain seated at all times.

### Biochemical analysis and salivary technique

The caffeine content of the coffee beverages was determined using high performance liquid chromatography (HPLC) [49]. Fasting salivary samples for measurement of salivary gastrin, salivary cortisol and sAA were collected 15 min after the volunteers’ arrival. Immediately afterward, the volunteers consumed one of the four test coffee beverages, and salivary samples were collected at 15, 30, and 60 min after coffee consumption for gastrin and sAA evaluation and at 60, 120 and 180 min for cortisol evaluation.

Salivary samples were collected using the «Salivette» method (Sarstedt AG and Co, Germany). Before the collection of the salivary samples, volunteers washed their mouths well with clear water to avoid contamination of saliva samples with food components and to avoid activation of salivary flow or protein production by gustatory stimuli [44]. Then, they were asked to remove the cotton from the tube and to move it around in a circular pattern for approximately 1 min to collect saliva from all glands [44]. The tubes were stored immediately at – 20 °C.

Salivary gastrin was measured with an ELISA immunoenzymic test (Abcam ltd, UK), sAA with kinetic enzyme assay kit (Salimetrics, UK) and salivary cortisol with an ELISA immunoenzymic test (Salimetrics, UK). All biochemical measurements were performed at Agricultural University of Athens.

### Anthropometric and blood pressure (BP) measurements

The body weight and height of volunteers were measured, and the BMI was calculated. Body composition analysis was performed using the bioelectrical impedance method (Tanita BC-408).

BP was measured at the beginning and end (180 min) of each intervention with a sphygmomanometer 3 times, with a 2 min interval between each measurement and with each participant being seated after 15 min of relaxation. The final value used was the mean of the three measurements.

### Dietary intake and physical activity analysis

Dietary intake was assessed with the use of a 24-h recall at every visit, for evaluation of the last 24 h dietary intake; a semi-quantitative food frequency questionnaire (FFQ), for evaluation of long-term dietary intake [50]; and a semi-quantitative modified FFQ that included only foods and beverages containing caffeine, for evaluation of the daily intake of caffeine (including all varieties of coffee, both caffeinated and decaffeinated, teas, caffeinated sodas, energy drinks, and foods/beverages containing chocolate). Dietary intake was analysed using the Diet Analysis Plus software (ESHA Research, OR, USA). Caffeine intake was calculated using the United States Department of Agriculture National Nutrient Database for Standard Reference and composition information from the food industry.

Physical activity was assessed with the use of a physical activity questionnaire [Harokopio Physical Activity Questionnaire (HPAQ)] [51] for evaluation of the weekly and daily energy expenditure. The physical activity questionnaire was analyzed using the metabolic equivalent of activities [52].

### Psychometric evaluations

Participants’ psychological state was evaluated with the use of 1. the Zung self-rating anxiety scale, which consisted of 20 questions, aimed to assess how the individual feels at the time of questioning [53], and was collected at baseline and 15, 30 and 60 min post-coffee consumption; 2. the Zung self-rating depression scale [54], collected at baseline; 3. the PSS-14 perceived stress questionnaire, which was validated for the Greek population [55] and collected at baseline; and 4. a self-reported scale questionnaire, which consisted of 29 questions with a score of 1 = not at all to 10 = very, evaluating reactions/symptoms, collected at baseline and at 15, 30, 60, 120 and 180 min post-coffee consumption. The self-reported scale questionnaire was developed by the clinical psychologist of our team and was used to assess GI symptoms and the psychological state of participants. [Questions: 1. I feel tremble; 2. I feel calm/relaxed; 3. I feel tension; 4. I feel that I have a migraine/headache; 5. I feel that my heartbeat is fast/racing; 6. I feel irritated/nervous; 7. I feel abdominal discomfort; 8. I feel stomach discomfort; 9. I feel abdominal pain; 10. I feel stomach ache; 11. I feel constipated; 12. I feel that coffee improves my bowel function; 13. I feel abdominal bloating/distension; 14. 1 feel like vomiting; 15. I feel dyspepsia; 16. I feel that coffee helps me with digestion; 17. I feel heartburn; 18. I feel that I am having gastroesophageal reflux; 19. I feel more energetic; 20. I feel intense fatigue; 21. I feel that I have difficulty concentrating; 22. I feel that my concentration is increased; 23. I feel vigilant; 24. I feel that my memory is better; 25. I feel that my memory is worse; 26. I feel mentally alert; 27. I feel that my mood is better; 28. I feel that my mood is worse; 29. I feel stressed/under pressure].

### Statistical analysis

Results are presented as the mean ± SEM. Differences between men and women in the volunteers’ descriptive characteristics were tested with a *t*-test for independent samples and the non-parametric Mann–Whitney test, depending on normality of the variables’ distribution. Normality was tested using the Kolmogorov-Smirnov test, as well as graphical methods (e.g., Q-Q plots) for the continuous variables. Calculations of areas under the curve (AUC) for salivary gastrin, salivary cortisol and sAA were based on the trapezoid rule. Differences in gastrin, sAA and cortisol concentrations, BP and stress scores between and among the 4 test coffee beverages, at the different time points and between genders were tested using repeated measures ANOVA with two within-subjects factors, type of coffee (hot instant, cold instant, cold espresso and hot filtered coffee) and time (0, 15, 30, 60 min and 0, 60, 120, 180 min respectively) and gender as an among-subject factor. Because gender and the respective interactions were not significant in all cases, except systolic BP, they were removed from the model. Moreover, for all parameters, one-way repeated measures ANOVAs were used to investigate differences between the types of coffee for each time point and for each type of coffee during the different time points. Multiple comparisons between the interventions were tested using the Tukey test. Differences between categorical data were tested using the chi-squared test. All statistical analyses were performed by SPSS (version 20.0, Chicago, IL, USA). The study had 80 % power (α = 0.05) to detect differences between dietary groups of 12.6 pg/ml in gastrin and 0.2 μg/ml in cortisol. For all tests, a *P*-value of less than 0.05 was considered to be statistically significant. Reporting of the study conforms to CONSORT-revised along with references to CONSORT-revised and the broader EQUATOR guidelines [56].

## Results

Subjects’ characteristics at baseline are described in Table 1. Body weight remained stable throughout the study. All volunteers consumed at least one cup of coffee per day with similar caffeine intake between men and women (Table 1). The majority of our volunteers had a high socioeconomic status and education level, with 42 % of the total sample having obtained a higher education degree and/or graduate school degree (Table 1). The scores on the psychometric questionnaires imply that our participants had moderately high perceived stress but normal self-reported anxiety and depression levels (Table 1).

**Table 1:** Volunteers’ descriptive characteristics at baseline (*n* = 40)

Characteristic	Total sample (*n* = 40)	Men (*n* = 20)	Women (*n* = 20)	P^1^
Age (years)	26.5 (23.0, 34.5)	31 ± 8.7	28 ± 7.8	NS
Body weight (kg)	70.9 ± 15.9	83.7 ± 11.0	58.1 ± 7.4	<0.001
Body Mass Index (kg/m^2^)	23.6 ± 3.5	25.7 ± 3	21.4 ± 2.4	<0.001
Body fat (%)	22.1 ± 6.9	20.1 ± 6.7	24.2 ± 6.7	NS
Lean body mass (kg)	54.8 ± 12.4	66.2 ± 5.9	43.3 ± 2.7	<0.001
Total body water (kg)	40.1 ± 9.1	48.5 ± 4.3	31.7 ± 2	<0.001
Daily caffeine intake (mg)	109.6 (49.9, 156.9)	120.2 (47.3, 159.5)	99.0 (49.9, 156.9)	NS
Daily energy intake (kcal)	1931.4 (1678.6, 2745.6)	2625.2 ± 878.5	1885 ± 437.2	<0.001
Carbohydrates (%)	43.7 ± 9.6	41.1 ± 8.5	46.3 ± 10.1	NS
Protein (%)	17.0 (13.5, 20)	17.8 ± 5.7	16.9 ± 4	NS
Fat (%)	37.9 ± 9.3	40.2 ± 8.1	35.7 ± 10.1	NS
Energy expenditure- HPAQ (kcal)	2400.0 (2070.0, 3128.0)	3061.6 ± 579.6	2070.0 (1643.5, 2203.5)	<0.001
Score of the PSS-14 Questionnaire (0–56)	31.8 ± 5.5	32.1 ± 4.3	31.6 ± 6.6	NS
Score of the Zung self-rating anxiety scale questionnaire (20–80)	30.9 ± 4.6	30.9 ± 5.3	31 ± 3.9	NS
Score of the Zung self-rating depression scale questionnaire (20–80)	33.8 ± 6.5	33.2 ± 6.5	34.4 ± 6.6	NS
Systolic blood pressure (mmHg)	122.6 ± 14.4	128.2 ± 12.3	117 ± 14.4	0.012
Diastolic blood pressure (mmHg)	79.4 ± 10.3	83.3 ± 5.8	75.5 ± 12.3	0.015

Data are Means ± SEM, or Median (1^st^ tertile, 3^rd^ tertile)

Abbreviations: *NS* not statistically significant

*P*-values < 0.05 were considered as significant

There was no significant main effect of gender and no gender x coffee interaction for salivary gastrin, salivary cortisol, sAA and psychometric evaluations, and therefore, our results are presented for the entire sample.

There was no main effect of coffee or coffee x time or time interactions for self-reported anxiety levels (Table 2). Moreover, no significant differences were found between the types of coffee at any time point from the self-reported scale questionnaire, which was developed for the purposes of our study and was used to evaluate reactions/symptoms. Volunteers reported an average score of 1.0 out of 10 for all the questions pertaining to negative GI symptoms (i.e., abdominal discomfort, bloating, dyspepsia, and heartburn), chronic stress and negative feelings and a score of 9.0 out of 10 for all the questions pertaining to positive feelings (Data not shown).

**Table 2:** Biochemical indices, self-reported anxiety levels^1^ at baseline and after coffee consumption in healthy volunteers in non-stressful conditions (*n* = 40)

	Cold espresso	Filter coffee	Cold instant coffee	Hot instant coffee	P^A 2^
Biochemical indices					
Salivary cortisol (mcg/dL)					
Baseline	0.31^a^ ± 0.03	0.32^a^ ± 0.02	0.32^a^ ± 0.03	0.30^a^ ± 0.02	NS
60 min	0.24^b^ ± 0.02	0.22^b^ ± 0.01	0.25^b^ ± 0.02	0.23^b^ ± 0.02	NS
120 min	0.23^bc^ ± 0.02	0.20^bc^ ± 0.01	0.20^bc^ ± 0.02	0.21^b^ ± 0.02	NS
180 min	0.19^c^ ± 0.01	0.17^c^ ± 0.01	0.17^c^ ± 0.01	0.18^b^ ± 0.01	NS
P^B 3^	<0.001	<0.001	<0.001	<0.001	
iAUC	11.25 ± 1.35	11.10 ± 1.10	13.41 ± 1.46	11.25 ± 0.98	NS
Salivary alpha-amylase (U/ml)					
Baseline	46.53^a^ ± 4.76	49.07^a^ ± 5.44	49.07^a^ ± 5.04	52.69^a^ ± 6.17	NS
15 min	58.93^aΑΒ^ ± 7.05	49.50^aΑ^ ± 5.23	70.27^bΒ^ ± 8.97	53.63^aΑΒ^ ± 5.94	0.030
30 min	94.50^bAB^ ± 10.94	74.81^bA^ ± 7.76	100.44^cB^ ± 11.19	83.41^bAB^ ± 8.67	0.024
60 min	103.54^bB^ ± 10.49	80.45^bA^ ± 8.77	97.35^cAB^ ± 10.44	86.61^bAB^ ± 8.91	0.034
P^B^	<0.001	<0.001	<0.001	<0.001	
iAUC	1019.29^ab^ ± 139.47	675.75^a^ ± 83.89	1214.79^b^ ± 167.35	772.67^a^ ± 89.74	<0.001
Salivary gastrin (pg/ml)					
Baseline	6.93^a^ ± 1.72	10.54^a^ ± 1.82	9.12^a^ ± 2.18	5.17^a^ ± 1.03	NS
15 min	51.18^c^ ± 5.36	47.53^b^ ± 5.93	60.33^b^ ± 10.40	62.95^c^ ± 8.98	NS
30 min	27.34^b^ ± 4.30	16.06^a^ ± 2.75	21.67^a^ ± 5.61	21.10^b^ ± 3.44	NS
60 min	10.46^a^ ± 3.19	7.74^a^ ± 1.88	12.64^a^ ± 3.43	6.45^ab^ ± 1.35	NS
P^B^	<0.001	<0.001	<0.001	<0.001	
iAUC	994.66 ± 74.03	730.16 ± 75.34	1377.81 ± 190.70	1251.22 ± 148.86	NS
Score of the Zung self-reported anxiety scale questionnaire (20–80)					
Baseline	28.6 ± 0.7	28.9 ± 0.7	28.3 ± 0.6	27.8 ± 0.7	NS
15 min	28.8 ± 0.6	28.8 ± 0.7	28.8 ± 0.7	27.6 ± 0.6	NS
30 min	29.5 ± 0.7	28.5 ± 0.6	29.2 ± 0.7	27.9 ± 0.6	NS
60 min	29.0 ± 0.6	28.8 ± 0.6	28.9 ± 0.6	28.1 ± 0.6	NS
P^B^	NS	NS	NS	NS	

Data are Mean ± SEM

Abbreviations: *NS* not statistically significant, *iAUC* incremental area under the curve calculated with the trapezoid rule. Means that have no superscript in common are significantly different from each other

*P*-values <0.05 were considered as significant

^1^Score of the Zung self-reported anxiety scale questionnaire (20–80) (Reference [45])

^2^P^A^ describes differences between coffee types at a specific time point

^3^P^B^ describes differences between time points for every coffee type

**Fig. 1:**
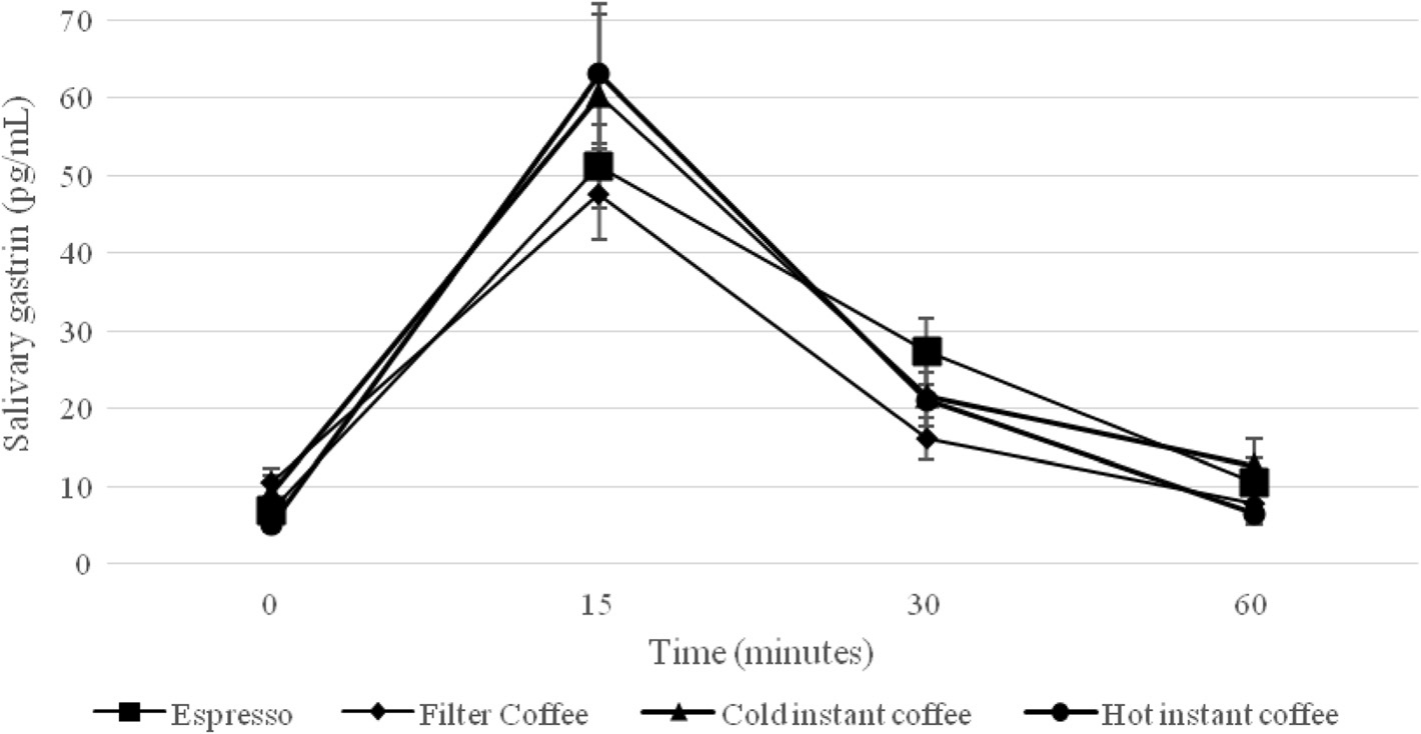
Salivary gastrin concentrations per time and per type of coffee in healthy participants under non-stressful conditions (*n* = 40). Data are means ± SEM. *P-values* < 0.05 were considered as significant

**Fig. 2:**
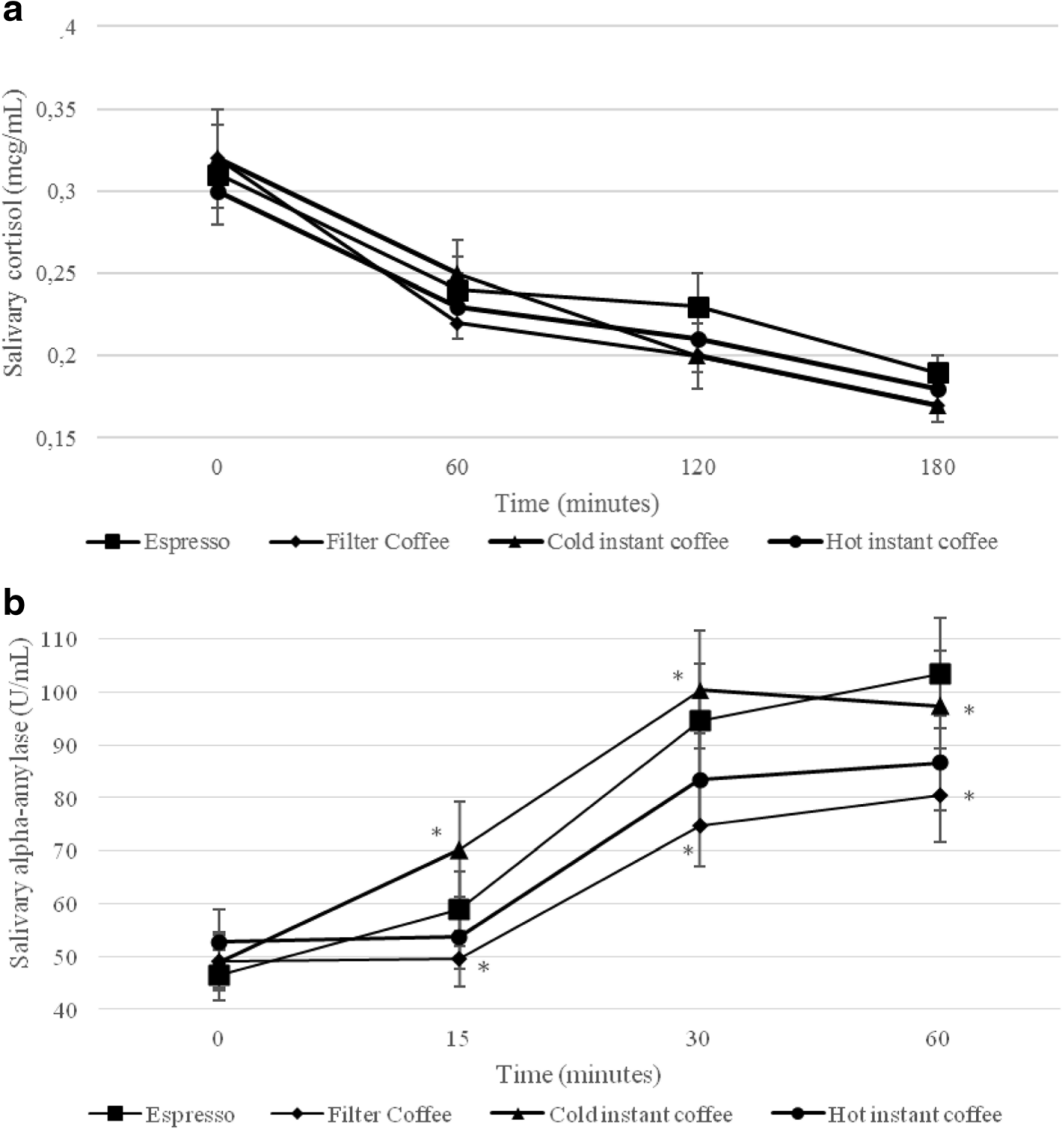
Salivary cortisol (**a**) and salivary alpha-amylase (**b**) concentrations per time and per type of coffee in healthy participants under non-stressful condition (*n* = 40). Data are means ± SEM. An asterisk indicates a statistically significant difference between the test coffee groups (*P* < 0.05)

At baseline, salivary gastrin concentrations were not different among the coffee types (Table 2, Fig 1). We found no significant main effect of coffee or coffee x time interaction, but did find a significant time interaction (*P* < 0.01) for salivary gastrin. At baseline, the salivary cortisol concentrations were not significantly different among coffee types (Table 2, Fig 2a). We found no significant main effect of coffee or coffee x time interaction, but did find a significant time interaction (*P* < 0.01) for salivary cortisol. At baseline, sAA concentrations were not different among coffee types (Table 2, Fig 2b). We found a significant main effect of coffee (*P* = <0.001), a significant coffee x time interaction (*P* = 0.003) and a significant time interaction (*P* < 0.001) for sAA. sAA concentrations increased significantly after consumption of all test coffees (*P* < 0.001; Table 2, Fig 2b), peaked at 30 min, and did not significantly differ between 30 and 60 min post-coffee consumption (Table 2). At 15, 30, and 60 min post-coffee consumption, the sAA concentrations were significantly higher after the consumption of cold instant coffee compared to filter coffee (*P* < 0.05; Table 2), cold instant coffee did not differ from hot instant coffee, and filter coffee did not differ from cold espresso (Table 2; Fig 2b). Instant coffee had a significant higher iAUC for sAA than did filter coffee (*P* < 0.001) and hot instant coffee (*P* = 0.007), without differences between cold instant coffee and cold espresso or between filter coffee and hot instant coffee or cold espresso.

Our participants were normotensives at baseline (Table 1). Coffee consumption increased BP within the healthy physiological levels at the end of the experimental periods (Table 3), but without differences between coffee types. There was a main effect of gender (*P* < 0.001) and time (*P* < 0.001) only for systolic BP (Table 3), but without a significant main effect of coffee, coffee x time, coffee x gender or gender x time interactions. There was no significant main effect of a gender, coffee, or gender x coffee, or coffee x time interactions, but there was a significant time interaction (*P* = 0.010; Table 3) for diastolic BP.

**Table 3:** Arterial blood pressure at baseline and after coffee consumption in healthy volunteers in non-stressful conditions (*n* = 40)

	Cold espresso	Filter coffee	Cold instant coffee	Hot instant coffee	P^A 1^
Arterial Blood Pressure (mmHg)					
Systolic Blood Pressure (mmHg)					
Baseline	116.67^a^ ± 1.54	118.21^a^ ± 1.95	116.74^a^ ± 1.95	118.53^a^ ± 1.64	NS
End	120.03^b^ ± 1.76	121.17^b^ ± 1.68	121.34^b^ ± 1.80	122.62^b^ ± 1.87	NS
P^B 2^	0.009	0.008	NS	0.007	
Systolic Blood Pressure (mmHg) in Men (*n* = 20)					
Baseline	120.70^a^ ± 1.81	122.62^a^ ± 2.65	123.07^a^ ± 2.00	122.88^a^ ± 2.07	NS
End	115.95^b^ ± 2.57	115.20^b^ ± 1.96	116.65^b^ ± 2.03	118.81^b^ ± 2.55	NS
P^B^	0.031	0.048	0.046	0.030	
Systolic Blood Pressure (mmHg) in Women (*n* = 20)					
Baseline	112.18^a^ ± 2.08	111.05^a^ ± 2.09	112.80^a^ ± 2.89	113.95^a^ ± 2.16	NS
End	115.95^b^ ± 2.57	115.20^b^ ± 1.96	116.65^b^ ± 2.03	118.81^b^ ± 2.55	NS
P^B^	0.031	0.048	0.046	0.030	
Diastolic Blood Pressure (mmHg)					
Baseline	75.27^a^ ± 1.12	77.09^a^ ± 1.35	77.33^a^ ± 1.34	78.15^a^ ± 1.47	NS
End	79.54^b^ ± 1.44	79.05^b^ ± 1.06	79.63^b^ ± 1.15	80.19^b^ ± 1.38	NS
P^B^	<0.001	0.049	0.019	NS	
Diastolic Blood Pressure (mmHg) in Men (*n* = 20)					
Baseline	74.90^a^ ± 1.42	76.63^a^ ± 1.72	76.85^a^ ± 1.48	78.44^a^ ± 1.38	NS
End	78.42^b^ ± 1.77	79.83^b^ ± 1.22	79.18^b^ ± 1.30	80.86^b^ ± 1.35	NS
P^B^	0.022	0.034	0.017	NS	
Diastolic Blood Pressure (mmHg) in Women (*n* = 20)					
Baseline	75.33^a^ ± 1.73	78.03^a^ ± 2.00	75.67^a^ ± 1.82	77.23^a^ ± 2.63	NS
End	80.92^b^ ± 2.19	79.43^b^ ± 1.91	78.53^b^ ± 1.70	80.00^b^ ± 2.47	NS
P^B^	<0.001	NS	0.005	NS	

Data are Mean **±** SEM

Abbreviations: *NS* not statistically significant

Mean that have no superscript in common are significantly different from each other. *P-values* <0.05 were considered as significant

^1^P^A^ describes differences between coffee types at a specific time point

^2^P^B^ describes differences between time points for every coffee type

## Discussion

The main findings of this study were that in healthy, daily coffee consumers, acute consumption of different types of coffee (instant coffee, espresso and filter coffee), containing equal amounts of caffeine 1. significantly increased sAA activity without a concomitant increase in salivary cortisol, implying a non-gender specific activation of the SNS in non-stressful conditions and also indicating that coffee consumption may have some anti-stress properties; 2. temporarily increased salivary gastrin concentrations, without differences among coffees, but had no effect on self-reported GI symptoms or other psychometric measures; and 3. all coffees significantly increased BP, but within the healthy physiological levels.

In our study, we found that salivary gastrin concentrations were only temporarily increased after coffee consumption, independent of coffee temperature [22], which is in agreement with some studies [15–19, 57], but not all [18, 20, 21]. In our study, the salivary gastrin values were comparable to plasma gastrin levels reported in other studies [22]. Moreover, contrary to previous reports [33], we did not find an interrelationship between salivary gastrin and salivary cortisol in healthy individuals in non-stressful conditions, indicating that salivary gastrin concentrations possibly follow the behaviour of cortisol only under physiological stress, as in the case of intense exercise. Furthermore, the discrepancy between increased salivary gastrin values and the lack of effect of coffee consumption on self-reported GI disturbances/symptoms in healthy, daily coffee consuming individuals without any previous history of GI problems is in agreement with others measuring plasma gastrin [6, 33, 58]; this finding indicates that GI disturbances are not coffee or gastrin related, but possibly associated with medium to intense physiological and/or psychological stress, as has been reported in both clinical trials and population studies [2, 9–11]. Furthermore, it seems that the interrelationship between GI symptoms, such as dyspepsia and abdominal pain, and increased plasma gastrin concentrations after coffee consumption depends on the individual characteristics and may only be relevant to a minority of individuals who are sensitive to caffeine [21, 59].

Cortisol is one of the most important stress hormones in humans, and increased basal levels of cortisol are considered to be a valid marker for sustained activation of the HPA axis. In our study, coffee consumption did not affect salivary cortisol concentrations in men or in women, which is in agreement with some studies [60–62], but not all [25–27, 29, 63]. Our results on the lack of a coffee effect on salivary cortisol may also be because of the non-stressful conditions and minimal anxiety levels of our subjects, as explored during the pre-enrolment screening of our study. Another study investigating the relationships between salivary cortisol profiles, job stress, work load and health in health care workers showed that coffee accounted for 8.4 % of the cortisol decline during the day and 15.2 % of the cortisol levels in the evening (2200 h) [60]. Another study, however, found that healthy adults taking repeated doses of caffeine during the day had increased cortisol response to caffeine during the afternoon hours, and the effect was gender dependent; however, in those consuming caffeine on a daily basis, the cortisol responses to caffeine were reduced but not eliminated, independently of gender [25]. Another study showed increased cortisol after coffee consumption only during an examination period compared to periods without examination [64]. Lastly, another study showed that caffeine administration in combination with an engaging non-stressful task decreased salivary cortisol in healthy, young, male, daily caffeine consumers [35]. The differences noted between studies may also be explained by the fact that salivary cortisol responses to caffeine intake in non-stressful conditions or diverse acute challenge paradigms show large intra- and inter-individual variability [65].

In our study, all coffee types, independently of gender, increased sAA activity with a significant main effect of coffee, coffee by time and time interactions. Cold instant coffee increased sAA significantly more than did filter coffee and hot instant coffee; however, cold instant coffee did not differ from cold espresso, and filter coffee did not differ from cold espresso or from hot instant coffee. These findings indicate that the temperature of the coffee beverage may affect sAA activity, but this has not been reported previously in the literature.

It has been shown that sAA activity shows a distinct diurnal profile pattern with a pronounced decrease within 60 min after awakening and a steady increase of activity during the course of the day [43]. Our results are in partial agreement with some studies showing that caffeine intake in habitual caffeine consumers significantly increases sAA activity [34, 66]. Furthermore, another study showed that caffeine intake, was the only statistically significant predictor of higher sAA levels among nurses at a paediatric intensive care unit [67]. Contrary to our findings, another study showed that caffeine intake in combination with an engaging non-stressful task did not affect sAA activity in healthy young male daily caffeine consumers [35]. However, due to the limited available literature, it is difficult to explain our sAA findings in regards to the sAA response to different coffee types. sAA activity may indicate the activation of the SNS to a challenge. However, when there is no parallel activation of cortisol, increased sAA activity may also indicate that the enzyme is a soothing or relaxation index [45]. Furthermore, our results on the lack of an effect of coffee consumption on perceived stress and the other psychometric measures, along with the lack of effect on salivary cortisol and increased sAA activation, may be consistent with coffee possessing some anti-stress properties.

In our study, we found that coffee consumption significantly increased BP, within the healthy physiological levels, in a gender specific manner in normotensive healthy individuals 3 h post-consumption, without differences between coffee types; which may be partially explained by SNS activation. Our results are in agreement with studies showing a temporary hypertensive coffee effect [35, 64, 68–70], even after small doses of consumed caffeine (80 mg/day) [31]. However, other studies have shown that the type of coffee may have a different impact on BP suggesting that boiled coffee may cause a higher increase in BP compared to filtered coffee, whereas instant coffee and decaf do not affect BP, after daily consumption of large coffee doses (>5 cups) for several days and/or weeks [71, 72]. The results of a meta-analysis of 6 prospective studies showed a caffeine dose-dependent positive association (J-shape effect) between coffee consumption and risk of hypertension, with the risk slightly increasing due to a small to medium consumption of 1–3 cups of coffee per day; however, consumption of 3 or more cups of coffee per day was not associated with a risk of hypertension [73]. Contrary to the aforementioned results, clinical trials with >2 weeks duration showed no association between coffee consumption and BP after daily consumption of medium to large coffee doses [74]. Similarly, the results from a meta-analysis of 10 clinical trials and 5 prospective trials showed that chronic coffee consumption had no significant effects on BP and hypertension risk, but the authors also noted that the available data are still of low quality for drawing safe conclusions [75].

This study is the first, to our knowledge, to investigate the effects of different types of coffee on self-reported GI symptoms, salivary gastrin, stress indices such as salivary cortisol and sAA, self-reported anxiety levels, and BP in healthy individuals (men and women) in non-stressful conditions. However, some limitations should be kept in mind when considering the results. Concerning salivary gastrin measurements, there is only one study in the literature showing a positive correlation between the gastrin measured in plasma and saliva (rho = 0.899, *P* < 0.001) [13] and further studies are needed to confirm these findings. As aforementioned, in our study the salivary gastrin values were comparable to plasma gastrin levels reported in other studies [22].

## Conclusion

The main findings of this study were that in healthy, daily coffee consumers, acute consumption of different types of coffee (instant coffee, espresso and filter coffee) containing equal amounts of caffeine 1. caused a significant activation of the SNS in non-stressful conditions by increasing sAA concentrations, without concomitant increases in salivary cortisol and without gender specificity, implying that coffee consumption may have some anti-stress properties; 2. temporarily increased salivary gastrin concentrations, without differences among coffees, but had no effect on self-reported GI symptoms or other psychometric measures; and 3. increased significantly BP, within the healthy physiological levels, without differences between coffee types.

The present results are intriguing and present more questions than answers with regard to the relationship between coffee consumption and GI function, stress, sAA activity, and BP. Nonetheless, these findings suggest that sAA be considered a valuable biomarker in biobehavioral studies on the health effects of coffee. In conclusion, the present study showed that acute coffee consumption (instant coffee, espresso, filter coffee) increased sAA and BP, thus activating the SNS, without effects on salivary cortisol or self-reported anxiety levels. Long-term clinical trials are needed to confirm our results and also to explore the coffee-mediated activation of the SNS and its effect on the gut-brain axis.

## References

[1] Holtmann G, Talley NJ (2014). The stomach-brain axis. Best Pract Res Clin Gastroenterol.

[2] Konturek PC, Brzozowski T, Konturek SJ (2011). Stress and the gut: pathophysiology, clinical consequences, diagnostic approach and treatment options. J Physiol Pharmacol.

[3] El Ansari W, Oskrochi R, Haghgoo G (2014). Are students' symptoms and health complaints associated with perceived stress at university? Perspectives from the United Kingdom and Egypt. Int J Environ Res Public Health.

[4] Rubach M, Lang R, Seebach E, Somoza MM, Hofmann T, Somoza V (2012). Multi-parametric approach to identify coffee components that regulate mechanisms of gastric acid secretion. Mol Nutr Food Res.

[5] Di Mario F, Goni E (2014). Gastric acid secretion: changes during a century. Best Pract Res Clin Gastroenterol.

[6] Shimamoto T, Yamamichi N, Kodashima S, Takahashi Y, Fujishiro M, Oka M (2013). No association of coffee consumption with gastric ulcer, duodenal ulcer, reflux esophagitis, and non-erosive reflux disease: a cross-sectional study of 8,013 healthy subjects in Japan. PLoS ONE.

[7] Saab S, Mallam D, Cox GA, Tong MJ (2014). Impact of coffee on liver diseases: a systematic review. Liver Int.

[8] Chen TS, Chang FY (2013). Elevated serum dopamine increases while coffee consumption decreases the occurrence of reddish streaks in the intact stomach. J Gastroenterol Hepatol.

[9] De Giorgi F, Sarnelli G, Cirillo C, Savino IG, Turco F, Nardone G (2013). Increased severity of dyspeptic symptoms related to mental stress is associated with sympathetic hyperactivity and enhanced endocrine response in patients with postprandial distress syndrome. Neurogastroenterol Motil.

[10] Stanghellini V (1999). Relationship between upper gastrointestinal symptoms and lifestyle, psychosocial factors and comorbidity in the general population: results from the Domestic/International Gastroenterology Surveillance Study (DIGEST). Scand J Gastroenterol Suppl.

[11] Wang JH, Luo JY, Dong L, Gong J, Tong M (2004). Epidemiology of gastroesophageal reflux disease: a general population-based study in Xi'an of Northwest China. World J Gastroenterol.

[12] Power ML, Schulkin J (2008). Anticipatory physiological regulation in feeding biology: cephalic phase responses. Appetite.

[13] Ingenito A, Catelani C, Mercaldo A, d'Agata A, Cappelli G, Andreoli F (1986). Radioimmunoassay of gastrin in human saliva. European surgical research Europaische chirurgische Forschung Recherches chirurgicales europeennes.

[14] Richardson CT, Walsh JH, Hicks MI, Fordtran JS (1976). Studies on the mechanisms of food-stimulated gastric acid secretion in normal human subjects. J Clin Invest.

[15] Borger HW, Schafmayer A, Arnold R, Becker HD, Creutzfeldt W (1976). The influence of coffee and caffeine on gastrin and acid secretion in man (author's transl). Dtsch Med Wochenschr.

[16] Wright LF, Gibson RG, Hirschowitz RI (1977). Lack of caffeine stimulation of gastrin release in man. Proceedings of the Society for Experimental Biology and Medicine Society for Experimental Biology and Medicine.

[17] Cohen S, Booth GH (1975). Gastric acid secretion and lower-esophageal-sphincter pressure in response to coffee and caffeine. N Engl J Med.

[18] Coffey RJ, Go VL, Zinsmeister AR, DiMagno EP (1986). The acute effects of coffee and caffeine on human interdigestive exocrine pancreatic secretion. Pancreas.

[19] Boekema PJ, Samsom M, van Berge Henegouwen GP, Smout AJ (1999). Coffee and gastrointestinal function: facts and fiction. A review. Scand J Gastroenterol Suppl.

[20] Feldman EJ, Isenberg JI, Grossman MI (1981). Gastric acid and gastrin response to decaffeinated coffee and a peptone meal. JAMA.

[21] Van Deventer G, Kamemoto E, Kuznicki JT, Heckert DC, Schulte MC (1992). Lower esophageal sphincter pressure, acid secretion, and blood gastrin after coffee consumption. Dig Dis Sci.

[22] McArthur KE, Feldman M (1989). Gastric acid secretion, gastrin release, and gastric emptying in humans as affected by liquid meal temperature. Am J Clin Nutr.

[23] Nehlig A, Daval JL, Debry G (1992). Caffeine and the central nervous system: mechanisms of action, biochemical, metabolic and psychostimulant effects. Brain Res Brain Res Rev.

[24] James JE (2004). Critical review of dietary caffeine and blood pressure: a relationship that should be taken more seriously. Psychosom Med.

[25] Lovallo WR, Whitsett TL, al'Absi M, Sung BH, Vincent AS, Wilson MF (2005). Caffeine stimulation of cortisol secretion across the waking hours in relation to caffeine intake levels. Psychosom Med.

[26] Gavrieli A, Yannakoulia M, Fragopoulou E, Margaritopoulos D, Chamberland JP, Kaisari P (2011). Caffeinated coffee does not acutely affect energy intake, appetite, or inflammation but prevents serum cortisol concentrations from falling in healthy men. J Nutr.

[27] Lovallo WR, Farag NH, Vincent AS, Thomas TL, Wilson MF (2006). Cortisol responses to mental stress, exercise, and meals following caffeine intake in men and women. Pharmacol Biochem Behav.

[28] Sunram-Lea SI, Owen-Lynch J, Robinson SJ, Jones E, Hu H (2012). The effect of energy drinks on cortisol levels, cognition and mood during a fire-fighting exercise. Psychopharmacology (Berl).

[29] Lane JD, Pieper CF, Phillips-Bute BG, Bryant JE, Kuhn CM (2002). Caffeine affects cardiovascular and neuroendocrine activation at work and home. Psychosom Med.

[30] Kudielka BM, Kirschbaum C (2005). Sex differences in HPA axis responses to stress: a review. Biol Psychol.

[31] Farag NH, Whitsett TL, McKey BS, Wilson MF, Vincent AS, Everson-Rose SA (2010). Caffeine and blood pressure response: sex, age, and hormonal status. J Womens Health.

[32] Mort JR, Kruse HR (2008). Timing of blood pressure measurement related to caffeine consumption. Ann Pharmacother.

[33] Gritti I, Banfi G, Roi GS (2000). Pepsinogens: physiology, pharmacology pathophysiology and exercise. Pharmacol Res.

[34] Klein LC, Bennett JM, Whetzel CA, Granger DA, Ritter FE (2010). Caffeine and stress alter salivary alpha-amylase activity in young men. Hum Psychopharmacol.

[35] Klein LC, Whetzel CA, Bennett JM, Ritter FE, Nater UM, Schoelles M (2014). Caffeine administration does not alter salivary alpha-amylase activity in young male daily caffeine consumers. BMC Res Notes.

[36] Nater UM, Rohleder N (2009). Salivary alpha-amylase as a non-invasive biomarker for the sympathetic nervous system: current state of research. Psychoneuroendocrinology.

[37] van Stegeren A, Rohleder N, Everaerd W, Wolf OT (2006). Salivary alpha amylase as marker for adrenergic activity during stress: effect of betablockade. Psychoneuroendocrinology.

[38] Proctor GB, Carpenter GH (2007). Regulation of salivary gland function by autonomic nerves. Auton Neurosci.

[39] Nater UM, Rohleder N, Gaab J, Berger S, Jud A, Kirschbaum C (2005). Human salivary alpha-amylase reactivity in a psychosocial stress paradigm. Int J Psychophysiol.

[40] Rohleder N, Wolf JM, Maldonado EF, Kirschbaum C (2006). The psychosocial stress-induced increase in salivary alpha-amylase is independent of saliva flow rate. Psychophysiology.

[41] Schenkels LC, Veerman EC, Nieuw Amerongen AV (1995). Biochemical composition of human saliva in relation to other mucosal fluids. Crit Rev Oral Biol Med.

[42] Granger DA, Kivlighan KT, el-Sheikh M, Gordis EB, Stroud LR (2007). Salivary alpha-amylase in biobehavioral research: recent developments and applications. Ann N Y Acad Sci.

[43] Nater UM, Rohleder N, Schlotz W, Ehlert U, Kirschbaum C (2007). Determinants of the diurnal course of salivary alpha-amylase. Psychoneuroendocrinology.

[44] Rohleder N, Nater UM (2009). Determinants of salivary alpha-amylase in humans and methodological considerations. Psychoneuroendocrinology.

[45] Takai N, Yamaguchi M, Aragaki T, Eto K, Uchihashi K, Nishikawa Y (2004). Effect of psychological stress on the salivary cortisol and amylase levels in healthy young adults. Arch Oral Biol.

[46] Cleophas TJ, Zwinderman AH (2002). Crossover studies with continuous variables: power analysis. Am J Ther.

[47] Wiese JG, Shlipak MG, Browner WS (2000). The alcohol hangover. Ann Intern Med.

[48] Melzer K, Kayser B, Pichard C (2004). Physical activity: the health benefits outweigh the risks. Curr Opin Clin Nutr Metab Care.

[49] Madison BL, Kozarek WJ, Damo CP (1976). High-pressure liquid chromatography of caffeine in coffee. Journal Association of Official Analytical Chemists.

[50] Bountziouka V, Bathrellou E, Giotopoulou A, Katsagoni C, Bonou M, Vallianou N (2012). Development, repeatability and validity regarding energy and macronutrient intake of a semi-quantitative food frequency questionnaire: methodological considerations. Nutr Metab Cardiovasc Dis.

[51] Kollia M, Gioxari A, Maraki M, Kavouras SAA, Greece:, 2006. Development, validity and reliability of the Harokopio Physical Activity Questionnaire in Greek adults. Proceedings of the 8th Panhellenic Congress on Nutrition and Dietetics Beta Medical Publishing. 2006.

[52] Ainsworth BE, Haskell WL, Whitt MC, Irwin ML, Swartz AM, Strath SJ (2000). Compendium of physical activities: an update of activity codes and MET intensities. Med Sci Sports Exerc.

[53] Samakouri M, Bouhos G, Kadoglou M, Giantzelidou A, Tsolaki K, Livaditis M (2012). Standardization of the Greek version of Zung's Self-rating Anxiety Scale (SAS). Psychiatrike = Psychiatriki.

[54] Fountoulakis KN, Lacovides A, Samolis S, Kleanthous S, Kaprinis SG, St Kaprinis G (2001). Reliability, validity and psychometric properties of the Greek translation of the Zung Depression Rating Scale. BMC Psychiatry.

[55] Andreou E, Alexopoulos EC, Lionis C, Varvogli L, Gnardellis C, Chrousos GP (2011). Perceived Stress Scale: reliability and validity study in Greece. Int J Environ Res Public Health.

[56] Simera I, Moher D, Hoey J, Schulz KF, Altman DG (2010). A catalogue of reporting guidelines for health research. Eur J Clin Invest.

[57] Acquaviva F, DeFrancesco A, Andriulli A, Piantino P, Arrigoni A, Massarenti P (1986). Effect of regular and decaffeinated coffee on serum gastrin levels. J Clin Gastroenterol.

[58] Higdon JV, Frei B (2006). Coffee and health: a review of recent human research. Crit Rev Food Sci Nutr.

[59] Brazer SR, Onken JE, Dalton CB, Smith JW, Schiffman SS (1995). Effect of different coffees on esophageal acid contact time and symptoms in coffee-sensitive subjects. Physiol Behav.

[60] Harris A, Ursin H, Murison R, Eriksen HR (2007). Coffee, stress and cortisol in nursing staff. Psychoneuroendocrinology.

[61] Spindel ER, Wurtman RJ, McCall A, Carr DB, Conlay L, Griffith L (1984). Neuroendocrine effects of caffeine in normal subjects. Clin Pharmacol Ther.

[62] Zanoboni A, Zanoboni MW (1987). Effects of naloxone and coffee on anterior pituitary hormones. Drugs Exp Clin Res.

[63] Lovallo WR, Al'Absi M, Blick K, Whitsett TL, Wilson MF (1996). Stress-like adrenocorticotropin responses to caffeine in young healthy men. Pharmacol Biochem Behav.

[64] Shepard JD, al'Absi M, Whitsett TL, Passey RB, Lovallo WR (2000). Additive pressor effects of caffeine and stress in male medical students at risk for hypertension. Am J Hypertens.

[65] Kudielka BM, Hellhammer DH, Wust S (2009). Why do we respond so differently? Reviewing determinants of human salivary cortisol responses to challenge. Psychoneuroendocrinology.

[66] Bishop NC, Walker GJ, Scanlon GA, Richards S, Rogers E (2006). Salivary IgA responses to prolonged intensive exercise following caffeine ingestion. Med Sci Sports Exerc.

[67] Morrison WE, Haas EC, Shaffner DH, Garrett ES, Fackler JC (2003). Noise, stress, and annoyance in a pediatric intensive care unit. Crit Care Med.

[68] Farag NH, Vincent AS, McKey BS, Al'Absi M, Whitsett TL, Lovallo WR (2006). Sex differences in the hemodynamic responses to mental stress: Effect of caffeine consumption. Psychophysiology.

[69] Farag NH, Vincent AS, Sung BH, Whitsett TL, Wilson MF, Lovallo WR (2005). Caffeine tolerance is incomplete: persistent blood pressure responses in the ambulatory setting. Am J Hypertens.

[70] James JE (1994). Chronic effects of habitual caffeine consumption on laboratory and ambulatory blood pressure levels. J Cardiovasc Risk.

[71] van Dusseldorp M, Smits P, Lenders JW, Thien T, Katan MB (1991). Boiled coffee and blood pressure. A 14-week controlled trial. Hypertension.

[72] Noordzij M, Uiterwaal CS, Arends LR, Kok FJ, Grobbee DE, Geleijnse JM (2005). Blood pressure response to chronic intake of coffee and caffeine: a meta-analysis of randomized controlled trials. J Hypertens.

[73] Zhang Z, Hu G, Caballero B, Appel L, Chen L (2011). Habitual coffee consumption and risk of hypertension: a systematic review and meta-analysis of prospective observational studies. Am J Clin Nutr.

[74] Mesas AE, Leon-Munoz LM, Rodriguez-Artalejo F, Lopez-Garcia E (2011). The effect of coffee on blood pressure and cardiovascular disease in hypertensive individuals: a systematic review and meta-analysis. Am J Clin Nutr.

[75] Steffen M, Kuhle C, Hensrud D, Erwin PJ, Murad MH (2012). The effect of coffee consumption on blood pressure and the development of hypertension: a systematic review and meta-analysis. J Hypertens.

